# Styrene monomer as potential material for design of new optoelectronic and nonlinear optical polymers: density functional theory study

**DOI:** 10.1098/rsos.240014

**Published:** 2024-07-31

**Authors:** P. Noudem, D. Fouejio, C. D. D. Mveme, S. S. Zekeng

**Affiliations:** ^1^ Mechanic Materials and Complex Structures Laboratory, Department of Physics, Faculty of Science, University of Yaoundé I, P.O. Box 812, Yaoundé, Cameroon; ^2^ Materials Science Laboratory, Department of Physics,Faculty of Science, University of Maroua, P.O. Box 814, Maroua, Cameroon

**Keywords:** styrene, DFT, functionalization, NLO, insulator, NBO

## Abstract

Using density functional theory, we have studied the intrinsic properties of styrene. First, we determine the optimized structures, structural parameters and thermodynamic properties to make our simulations more realistic to experimental results and check the stability. Second, we investigate optoelectronic, electronic and global descriptors, transport properties of holes and electrons, natural bond orbital analysis, absorption and fluorescence properties. Finally, we study nonlinear optical (NLO) properties: first- and second-order hyperpolarizability, second and third-order optical susceptibilities, hyper-Rayleigh scattering hyperpolarizability, electro-optical Pockel effect, direct current Kerr effects and quadratic refractive index. The bandgap energy *E*_g_ = 5.146 eV and dielectric constant $\varepsilon_{r}=3.062$ show that styrene is a good insulator with an average electric field value of 4.43 × 10^8^ Vm^−1^. Thermodynamic findings show that our molecule is thermodynamically and chemically stable. Electron and hole reorganization energies of 0.393 and 0.295 eV, respectively, show that styrene is more favourable to hole transport than electron transport. Styrene is transparent with linear refractive index *n* = 1.750 and quadratic *n*_2_ = 1.748 × 10^−20^ m^2^ W^−1^. At the NLO, styrene has a non-zero value of $\beta_{HRS},$ which confirms the existence of first-order NLO activity. Globally the study shows that the styrene monomer is suitable for the architecture design of new polymer materials for NLO applications and optoelectronic by functionalization.

## 1. Introduction

Computation now plays a key role in materials innovation. It facilitates our understanding and design of materials, providing a comprehensive overview of their behaviour, from the Angstrom to the micrometre scale, and from the femtosecond to the millisecond [1,2]. For the understanding, prediction and design of materials, the density functional theory (DFT) method, which is an electronic structure method, can be used to predict with very good accuracy: (i) the fundamental properties of materials from their smallest constituents: atoms, unit cells and chemical bonds between atoms; and (ii) ground-state properties (ground-state energy and its derivatives, thermodynamics) as well as excited-state properties (optical absorption and emission) [1,3–5]. DFT is currently a mature and widely used method for material simulations [3,6–8]. With regards to the development of research into complex and non-complex materials, like nanocages [9], simulations are essential, as they enable materials to be assessed under pressure and temperature conditions that are difficult to access experimentally. They also make it possible to directly identify microscopic causes, as well as the origin of a macroscopic property [3]. Finally, one of the main advantages of simulations is that they can be reproduced, opened up and shared [3,10]. More recently, researchers have been experimenting with the use of machine learning to identify the descriptors that influence the performance of organic solar cells (OSCs) to design new small donor molecules suitable for improving OSCs performance [11,12].

The development and understanding of organic semiconductors have positioned this class of materials as the new electronics revolution of the early twenty-first century. Their applications span diverse fields, including flexible light sources, display devices, low-cost printed integrated circuits and plastic solar cells [13]. Organic semiconductors have led to applications in optoelectronic devices such as organic light-emitting diodes (OLEDs), organic field-effect transistors and organic photovoltaic solar cells [14–17]. In the field of nonlinear optical (NLO) properties, organic materials and organic semiconductors have several applications such as NLO devices, memory storage devices [18–20], optical communication, optical switching, laser technologies, dynamic image processing and optical computing [21–23]. In addition, organic materials offer numerous advantages over inorganic materials, such as high laser damage thresholds, high NLO susceptibilities, reduced dielectric constants, a wide transparency range, ultrafast response times and easier processing in the front line of NLO research [24,25]. In the same vein, some small organic compounds can also be used as models for the design of supramolecular systems, molecular logic gates, controllable switches and sensors, as well as in nanotechnology and signal processing [5,26–28].

In recent years, the design of new materials with high NLO response and good optoelectronic, transport and electronic properties has become the focus of most scientists and a major research topic worldwide. Many approaches have been developed including [29–32], the planar donor π-conjugated bridge acceptor (D-π-A) model, modification of the D-π-A structure [33,34], the use of halogen doping [32,35], alkali and super alkali metal doping [24], as well as organic dopants [36], organometallic design and complex formation [37], functionalization [38,39] and so on. One of the best approaches to designing this type of material is functionalization, an important process for modifying the physical and chemical properties of organic materials [40]. According to several researchers [2,4,5,38], functionalizing carbon chains is a suitable way of enhancing the electronic and optical properties of organic semiconductor compounds such as chromophores. According to Stadtmüller *et al.* [41] , chemical functionalization provides interesting possibilities for exploiting the tunability of structural and electronic properties of organic materials, which could lead to a new class of functional materials with applications in electronics and spintronics. In addition, functionalization serves as a powerful tool for designing new polymers and constructing macromolecular structures with predictable architectures from small organic molecules [42]. While several polymers are commonly recognized for their use in functionalizing organic materials for optical and optoelectronic applications, including polymethyl methacrylate (PMMA), polystyrene (PS) and polyacrylamide [38,42,43]. Our previous work focused on the study of methyl methacrylate (MMA) for optoelectronic and optical applications [4]. In this work, we are focusing on styrene monomer, which presents a credible alternative to MMA.

Styrene was first isolated in 1839 [44] and is one of the most widely used monomers with a variety of applications in the chemical industry to produce PS, acrylonitrile-butadiene-styrene rubber and many other polymers [45]. The advantages of styrene monomer and its derivatives that make them attractive compounds compared with other polymers are low cost and low density, durability and good resistance during processing, ease of processing and moulding, as well as other special PS characteristics, such as low moisture absorption and transparency. Indeed, the refractive index at 25°C of styrene monomer is *n* = 1.544, while PS offers high brightness with *n* = 1.592 and high transmission of all visible light wavelengths [46,47].

Nowadays, styrene monomer continues to be the focus of investigation for many research teams around the world owing to its numerous applications and uses [48,49]. PS and styrene monomers are widely used in various industries and are topical in many areas of research such as new effective commercial stabilizers [50], composite science and technology [51] for the manufacture and functionalization of transparent wood [52]. They are also used in the rubber industry [53], in electronics such as vacuum cleaners and telephone housings [47], in the food packaging industry [46,54,55], in ZnO/PS nanocomposites, in vinyl ester [52], in electronic devices [46,52] and in the design and synthesis of new organic semiconductors and optical materials [56].

In the midst of all these multiple and current applications of styrene monomer, we focus in this work, on the use of styrene monomer for the design of organic semiconductors, display devices, transparent and flexible electronics and NLO materials. In this respect, a prior understanding of the intrinsic behaviour of styrene monomers is essential to improve the quality of the design of new materials through styrene functionalization. Furthermore, the intrinsic behaviour of styrene monomer in terms of optics, electronics, thermodynamics and charge transport, before chemical reactions with other compounds, is not sufficiently documented in the literature, which in our view constitutes a lack of information that needs to be addressed. Indeed, to date, no theoretical studies were carried out on the determination of structural, electronic, optoelectronic, linear and NLO, thermodynamic, absorption and emission properties, as well as chemical reactivity descriptors, charge transfer and natural bond orbital (NBO) analysis of styrene monomer.

Therefore, this work aims to perform a DFT study of the electronic structure of styrene monomers. To this end, we will determine the above-mentioned electro-optical properties of styrene and highlight the advantages of styrene monomer over MMA monomer in the design of optoelectronic and optical devices through functionalization.

## 2. Methodology and computational details

Linear optical properties of styrene monomer are evaluated through physical parameters such as dipole moment (μ) , average polarizability ( $\bar{\alpha }$ ) and anisotropy (Δ*α*) and first-order susceptibility ( $\chi_{e}^{\left(1\right)}$ ), which are given by the following equations:


(2.1) $$\mu ={\left(\mu_{x}^{2}+\mu_{y}^{2}+\mu_{z}^{2}\right)}^{1/2},$$

(2.2) $$\bar{\alpha }=\frac{1}{3}\left(\alpha_{xx}+\alpha_{yy}+\alpha_{zz}\right),$$

(2.3) $$\Delta \alpha =\frac{1}{\sqrt{2}}{\left[{\left(\alpha_{xx}-\alpha_{yy}\right)}^{2}+{\left(\alpha_{yy}-\alpha_{zz}\right)}^{2}+{\left(\alpha_{zz}-\alpha_{xx}\right)}^{2}+6\left(\alpha_{xy}^{2}+\alpha_{xz}^{2}+\alpha_{yz}^{2}\right)\right]}^{1/2},$$

(2.4) $$\chi_{e}^{\left(1\right)}=\frac{1}{3}\left(\chi_{xx}+\chi_{yy}+\chi_{zz}\right).$$

For the characterization of the NLO behaviour, we calculate the first total hyperpolarizability, $\beta_{T}$ and the averaged second hyperpolarizability, $\bar{\gamma }$ of the monomer using the following equations:


(2.5) $$\beta_{T}={\left(\beta_{x}^{2}+\beta_{y}^{2}+\beta_{z}^{2}\right)}^{1/2},$$

(2.6) $$\bar{\gamma }=\frac{1}{5}\left[\gamma_{xxxx}+\gamma_{yyyy}+\gamma_{zzzz}+2\left(\gamma_{xxyy}+\gamma_{xxzz}+\gamma_{yyzz}\right)\right]$$

where $\beta_{x}$ , $\beta_{y}$ and $\beta_{z}$ are given by


(2.7) $$\beta_{i}=\beta_{iii}+\frac{1}{3}\sum_{i\neq j}\left(\beta_{ijj}+\beta_{jij}+\beta_{jji}\right);i,j=x,y,z.$$

Regarding the electronic behaviour of styrene, the adiabatic ionization potential (IP) and adiabatic electron affinity (EA) are important parameters for describing charge transport processes and molecular chemical stability. They are obtained from the following relationships [57–62]:


(2.8) $$\begin{array}{c} \text{IP}=E\left(M+\right)-E\left(0\right) \end{array},$$

(2.9) $$\begin{array}{c} EA=E\left(0\right)-E\left(M-\right) \end{array},$$

where E(M+) and E(M−)  are the energies of the molecule, obtained after optimization of its cation and anion states respectively. E(0) is the energy of the neutral compound taken in the ground state at the end of the optimization. The bandgap energy was calculated as follows:


(2.10) $$E_{g}=E_{LUMO}-E_{HOMO},$$

where $E_{HOMO}$ is the energy of the highest occupied molecular orbital and $E_{LUMO}$ the energy of the lowest unoccupied molecular orbital.

Optoelectronic properties of styrene monomer such as electric displacement (*D*), electric field in the material (*E*), relative permittivity of the material ( $\varepsilon_{r}$ ), induced polarization (*P*) and refractive index (*n*) were calculated using equations available in the literature [5,63]:


(2.11) $$E=\frac{\mu }{\overset{-}{\alpha }},$$

(2.12) $$P=\frac{\mu }{V}.$$

In addition, the relative permittivity $\varepsilon_{r}$ and the dielectric constant ε are given by $\varepsilon_{r}=1+\chi_{e}$ and $\varepsilon =\varepsilon_{0}\varepsilon_{r}$ . The refractive index is obtained using equation $n=\sqrt{\varepsilon_{r}}=\sqrt{1+\chi_{e}}$ .

Time-dependent DFT (TD-DFT) was used to study the excited states of styrene monomer including absorption and emission spectra, while DFT was used for the other properties. GaussView 6.0.16 software [64] was used for modelling and data visualization, while Gaussian 16 software [65] was used for all atomistic calculations, such as structural, thermodynamic, electronic, optoelectronic and NLO properties, as well as chemical reactivity descriptors, NBO and charge transport of styrene monomer. All calculations were performed in the gas phase at room temperature and standard pressure. Two functionals, B3LYP and ωB97XD were used. According to the literature, the B3LYP functional is suitable for studying the electronic, transport, NBO and thermodynamic properties of organic molecules [66–69]. Meanwhile, the ωB97XD functional is a corrected long-range hybrid functional that enables precise assessment of the optical and chemical quantum properties of organic molecules, as well as more realistic studies of excited states [10,35,66,70,71]. In this work, the ωB97XD functional will be used as a reference for the prediction of NLO properties, while the B3LYP will be the reference for the characterization of electronic, optoelectronic and transport properties. The basis set 6−311G(d,p) was used for all the calculations.

## 3. Results and discussion

### 3.1. Optimized structures

**Figure 1.**
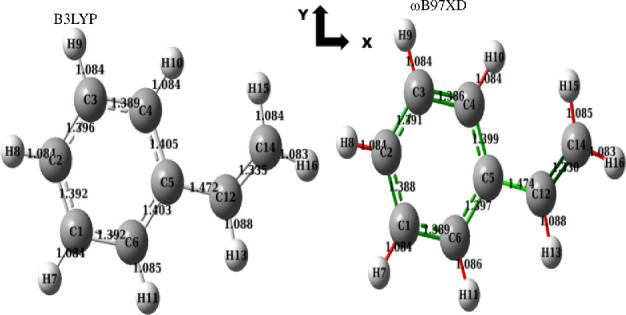
Optimized structures of the styrene monomer obtained using ω B97XD and B3LYP functionals.

The optimized structures using ωB97XD and B3LYP functionals of our styrene monomer are shown in figure 1. No negative frequencies were observed after the optimizations, which leads to the existence of local minima on the potential energy surface at the end of the optimizations [72] and therefore, the optimized styrene monomer is geometrically stable.

It can be seen from figure 1 that the only and main difference between optimization with the ωB97XD and B3LYP methods is at the C3−C4 bond. Indeed, optimization using ωB97XD leads to a C=C double bond between C3 and C4, while a delocalized bond is obtained using the B3LYP method.

All bond lengths and some selected valence angles of styrene monomer are collected in table 1. The structural parameters of styrene monomer were determined experimentally at 87 K in 2001 by Yasuda *et al*. [73].

**Table 1.** Bond lengths (in Å) and some selected angles (in °) of styrene monomer.

bond lengths (Å)						angles (°)			
designation	B3LYP	ωB97XD	EXP^a^	ΔB3LYP	ΔωB97XD	designation	B3LYP	ωB97XD	EXP^a^
C1–C2	1.392	1.388	1.388	0.003	0.000	C2–C1–C6	120.04	120.027	120.077
C1–C6	1.392	1.389	1.390	0.001	−0.001	C2–C1–H7	120.151	120.01	120.375
C1–H7	1.084	1.084	0.991	0.094	0.094	C6–C1–H7	119.808	119.873	119.544
C2–C3	1.396	1.391	1.392	0.003	−0.001	C1–C2–C3	119.453	119.511	119.432
C2–H8	1.084	1.084	1.009	0.075	0.075	C1–C2–H8	120.321	120.264	120.529
C3=C4	1.389	1.386	1.388	0.001	−0.001	C3–C2–H8	120.226	120.224	120.025
C3–H9	1.084	1.084	0.974	0.112	0.112	C2–-C3–C4	120.423	120.355	120.483
C4–C5	1.405	1.399	1.402	0.002	−0.002	C2–C3–H9	119.92	119.938	120.677
C4–H10	1.084	1.084	0.983	0.102	0.102	C4–C3–H9	119.657	119.705	118.840
C5–C6	1.403	1.397	1.395	0.006	0.001	C3–C4–C5	120.899	120.802	120.571
C5–C12	1.472	1.474	1.474	−0.001	0.000	C3–C4–H10	119.222	119.348	119.051
C6–H11	1.085	1.086	0.989	0.097	0.098	C5–C12=C14	127.6628	126.817	126.790
C12–H13	1.088	1.088	0.981	0.109	0.109	H13–C12=C14	117.9266	118.3775	117.868
C12=C14	1.335	1.330	1.325	0.008	0.004	C12=C14 H15	122.8434	122.5279	123.381
C14–H15	1.084	1.085	0.967	0.121	0.122	C12=C14 H16	120.8199	120.819	119.975
C14–H16	1.083	1.083	0.989	0.095	0.095	H15–C14–H16	116.3366	116.6517	116.549

^a^ Experimental values from Yasuda *et al*. [73].

We found good agreement by comparing our DFT findings with experimental results. Indeed, as shown in table 1, the B3LYP and ωB97XD functionals give the same values for many bond lengths and angles. Our DFT methods are precise for the determination of carbon–carbon bond lengths and angles, while we have some small discrepancies on the evaluation of C−H bond length.

ωB97XD functional is slightly more precise than B3LYP for some bonds and angles. Moreover, the maximum difference between the present theoretical values and the experimental values does not exceed 0.13 whatever the functional used. Furthermore, we can conclude that the functionals and basis sets used for this study are well chosen, and that the results obtained may be more realistic at the end of the atomistic simulations.

It appears from the values in table 2 that the styrene monomer exhibits a planar structure relative to the B3LYP and ωB97XD optimizations. All atoms and bonds are predominantly present in the (*x*,*y*) plane.

**Table 2.** Cartesians coordinates (in Å) of atoms of styrene monomer.

designation	B3LYP			ωB97XD		
coordinates	*x*	*y*	*z*	*x*	*y*	*z*
C1	−1.778	−1.044	0.000	−1.770	−1.042	0.049
C2	−2.261	0.261	0.000	−2.252	0.261	0.040
C3	−1.360	1.327	0.000	−1.355	1.322	−0.022
C4	0.009	1.090	0.000	0.010	1.085	−0.068
C5	0.514	−0.220	0.000	0.511	−0.221	−0.048
C6	−0.406	−1.279	0.000	−0.403	−1.277	0.002
H7	−2.468	−1.880	0.000	−2.461	−1.876	0.091
H8	−3.329	0.449	0.000	−3.319	0.449	0.074
H9	−1.728	2.347	0.000	−1.724	2.342	−0.041
H10	0.691	1.932	0.000	0.694	1.924	−0.133
H11	−0.036	−2.299	0.000	−0.032	−2.297	0.010
C12	1.953	−0.528	0.000	1.953	−0.523	−0.080
H13	2.186	−1.591	0.000	2.197	−1.568	−0.265
C14	2.972	0.335	0.000	2.952	0.335	0.108
H15	2.832	1.410	0.000	2.786	1.386	0.320
H16	3.997	−0.016	0.000	3.983	0.005	0.063

### 3.2. Optoelectronic properties

Determining and interpreting the optoelectronic properties of styrene monomer is important for understanding the optoelectronic properties of macromolecules based on the functionalization of certain chromophores by styrene. Optoelectronic properties provide information for characterizing light propagation in organic and inorganic media. They are also important to predict applications of studied materials in OSCs, ultrafast response and OLEDs, based on their ability to convert an optical signal into an electrical signal [4]. We have summarized in table 3, the obtained results of our DFT investigation through B3LYP functional.

**Table 3.** Average electric field (*E*), electric polarization (*P*), average electric susceptibility (*χ*_e_), relative dielectric constant (ε_r_), dielectric constant (*ε*), refractive index (*n*), electric displacement (*D*), electrical impermeability (*η*), coefficient of reflectivity (CR) of styrene monomer, obtained using B3LYP.

parameters	B3LYP
*E* (×10^9^ Vm^−1^)	0.443
*P* (×10^−2^ cm^−2^)	0.809
*D* (×10^−2^ cm^−2^)	1.202
ε (×10^−11^)	2.711
$\varepsilon_{r}$	3.062
$\chi_{e}$	2.062
*n*	1.750
*η*	0.327
CR (%)	7.400

The local electric field (*E*) results from the distribution of electric charges carried by the atoms within the molecule and applied to all charges in the molecular system. Depending on its intensity, it can influence the electrical and optical properties of the material. We obtained $E=0.443\times 10^{9}\;Vm^{-1}$ through the B3LYP; a value at least 13 times lower than that observed in MMA [4]. As a result, MMA monomer is a much better candidate than styrene monomer for the design of new high-field materials through functionalization. As regards, *P*, it is a measure of the distribution of electrical charges. It is created by the separation of positive and negative charges in the molecule, owing to the movement of electrons [25]. We obtained $P=0.802\times 10^{-2}cm^{-2}$ , a rather low value which highlights a weak charge distribution in the styrene monomer. Regarding, *D*, we obtained a value of $1.202\times 10^{-2}{cm}^{-2}$ , a value that reflects a low charge density in styrene. Comparing the *P* and *D* values of styrene monomer with those of MMA monomer under the same calculation conditions, we found that the electric polarization of MMA is 11 times greater than that of styrene. Similarly, the electric displacement of MMA is 12 times greater than that of styrene monomer. Styrene is therefore not a potential material for piezoelectric and pyroelectric applications.

As regards $\varepsilon_{r}$ , it characterizes the material’s ability to store electrical energy and enables us to discuss potential applications of materials as insulators [4], styrene monomer exhibits a dielectric behaviour of $\varepsilon_{r}=3.062$ . This is a significant value, making it a good insulator when compared with good insulators such as poly(ethylene terephthalate) ( $\varepsilon_{r}=3.0$ ). In addition, styrene monomer has a slightly higher electrical energy storage capacity than MMA monomer in its *cis* ( $\varepsilon_{r}=2.810$ ) and *trans* ( $\varepsilon_{r}=2.993$ ) forms [4]. Regarding the refractive index, we obtained *n* = 1.750 at the B3LYP level. This value is 13% higher than the experimental value of 1.544 obtained at 25°C [42,47]. This value reflects a transparency comparable to that of glass with a refractive index of 1.5 [74,75]. By comparing with *cis*-MMA (*n* = 1.676) and *trans*-MMA (*n* = 1.730) at the B3LYP level, we can conclude that styrene monomer offers high transparency and the same ease of light propagation as MMA. Styrene monomer is therefore a good candidate for the design of transparent polymers through chromophore functionalization. Finally, when it comes to materials for optoelectronics and data transmission, such as optical fibers, highly transparent materials are the focus of researchers' attention, as they reduce reflections at the diopter level and enable better transmission of the light signal with little loss. We obtained a reflection coefficient of 7.40% in styrene monomer. Given that the reflection coefficients of common semiconductors are of the order of 30% [76], we conclude that there is about four times less loss in styrene than in common semiconductors during light signal transmission, making styrene a good potential functionalizer for the design of transparent semiconductors. Finally, the electrical impermeability of styrene is 0.327, a value very close to that of MMA monomer.

### 3.3. Charge mobility

Reorganization energy is a parameter that characterizes charge mobility in organic materials [77]. The reorganization energy of the electron (*E*_e_) as well as that of the hole (*E*_h_) can be calculated for an organic compound using the Marcus theory [78,79], through the following formulas:


(3.1) $$E_{e}=\left(E_{-1}^{q=0}-E\left(M-\right)\right)+\left(E_{0}^{q=-1}-E\left(0\right)\right),$$

(3.2) $$E_{h}=\left(E_{+1}^{q=0}-E\left(M+\right)\;\right)+\left(E_{0}^{q=+1}-E\left(0\right)\right).$$

where $E_{0}^{q=-1}$ and $E_{0}^{q=+1}$ are the energies of the neutral compound in the anionic and cationic states, respectively. E(0) is the energy of the neutral compound taken in the ground state at the end of optimization, whereas the energies $E_{+1}^{q=+1}$ and $E_{-1}^{q=-1}$ are obtained from the optimization of the cationic and anionic forms of the compound. $E_{-1}^{q=0}$ and $E_{+1}^{q=0}$ are the energies of the neutral form obtained from the optimized structures of the anion and cation, respectively. The integral charge carrier transfer coefficients of the electrons $t_{e}$ and holes $t_{h}$ are obtained from the following relations [60–62]:


(3.3) $$t_{e}=\frac{E_{LUMO+1}-E_{LUMO}}{2},$$

(3.4) $$t_{h}=\frac{E_{HOMO}-E_{HOMO-1}}{2}.$$

The findings of the transport properties of the studied compound, calculated using the B3LYP and ωB97XD functionals are given in table 4. We obtained electron and hole reorganization energies of 0.393 and 0.295 eV, respectively. These values indicate that holes reorganize faster than electrons in styrene. Generally, the literature indicates that low values of electron and hole reorganization energies indicate better rates of charge transport and carrier mobility [59]. Consequently, styrene monomer is more favourable for hole transport than for electron transport. Comparing these values with those for MMA monomer, it can be seen that charge carriers reorganize faster and require lower energies in styrene than in MMA. In addition, the reorganization energy of electrons in MMA is two times that of styrene, while that of holes is four to nine times that of styrene. Styrene monomer is therefore more suitable than MMA for the functionalization of new charge-transporting molecules. In addition, the integral charge transfer coefficient assesses the charge transport properties of an organic molecule. Higher values of integral charge transfer coefficients reflect better charge carrier mobility [80]. In our study, we obtained an integral charge transfer coefficient of 0.430 eV for electrons and 0.375 eV for holes.

**Table 4.** Reorganization energy (*E*_h_, *E*_e_) and integral charge transfer (*t*_e_, *t*_h_) of holes and electrons of styrene monomer calculated using the B3LYP and ωB97XD methods. (Values are given in eV.)

parameters	B3LYP	ωB97XD
*E*_e_	0.393	0.490
*E*_h_	0.295	0.380
*t*_e_	0.430	0.441
*t*_h_	0.375	0.402

Numerous current studies available in the literature on the problem of efficient charge transport in OSCs show that, in general, low values of reorganization energies promote or induce rapid movement of electrons and holes between the metal electrodes of OSCs [81–83]. To characterize the transport properties of styrene, we decided to compare its values with those of other materials recently published in the literature. Thus, in 2022, Janjua [84] designed and numerically studied new organometallic materials for hole and electron transport through halogen doping. The maximum electron and hole mobilities obtained were 0.198 and 0.201 eV, respectively. These values are 1.98 and 1.46 times smaller than those of the intrinsic styrene monomer. Interestingly, even in its neutral form, styrene despite being an insulator exhibits acceptable hole and electron transport properties. However, there are other molecules with transport parameters around *E*_e_ = 0.022 eV and *E*_h_ = 0.020 eV that enable the creation of OSCs with high fill factors and improved open-circuit voltage [81,85,86]. When comparing these values to those of styrene, it becomes evident that they are on average 12 to 15 times smaller than those of the styrene monomer. Styrene in its intrinsic form can thus serve as a basis for designing even more efficient molecules for rapid electron and hole transport, leading to enhanced OSC efficiencies.

### 3.4. Electronic properties and frontier molecular orbital analysis

Some electronic parameters of the styrene monomer were used to assess its electrical conduction properties. The ability of frontier orbitals to give up or receive electrons was also assessed. Parameters such as *E*_LUMO_, *E*_HOMO_, *E*_g_, IP, EA as well as the photon’s threshold wavelength (*λ*), Fermi energy (*E*_Fermi_) and fundamental gap (*E*_f_) were evaluated and reported in table 5. We obtained *E*_g_ = 5.146 eV, a very high value (>4 eV), so the electron has difficulty crossing this energy barrier. As a result, there are very few free electrons in the conduction band and the material has low electric conduction, making it an electrical insulator.

**Table 5.** *E*_HOMO,_ *E*_LUMO_, *E*_g_, *E*_Fermi_, *E*_f_, IP and EA of styrene monomer, obtained at the B3LYP and ωB97XD levels of theory. (Energy values are given in eV and wavelength in nm.)

parameters/method	B3LYP	ωB97XD
*E*_LUMO_	−1.154	0.772
*E*_HOMO_	−6.300	−8.279
*E*_g_	5.146	9.051
*λ*	240.954	137
*E*_Fermi_	−3.727	−3.753
IPv	8.252	8.401
EAv	−0.727	−0.878
*E*_f_ *v*	8.979	9.279
IPa	8.105	8.205
EAa	−0.532	−0.623
*E*_f_ *a*	8.637	8.828

The threshold wavelength of the photon that allows the electron to jump from the HOMO to the LUMO level is λ = 240.954 nm, while the Fermi energy level is *E*_Fermi_ = −3.727 eV. Fermi energy represents the average energy of an electron in a material at thermodynamic equilibrium. In styrene monomer, the Fermi level is below the middle of the band gap.

With regards to $E_{f}$ , which is another measure of electronic stability and a parameter well suited to describing the reactivity of a molecule [87], we recorded values of 8.637 and 8.979 eV for the vertical and adiabatic determination. Both values indicate the existence of high electronic stability in styrene, which confirms the insulating nature of styrene, because for broadband organic semiconductors, the fundamental gap is generally between 4 and 7 eV [2]. We can also observe that the fundamental gap is highest when determined in the adiabatic regime.

As far as the IP is concerned, it provides an estimate of the energy barrier to be crossed for the extraction of charge carriers in an organic material [88,89]. We obtained a value of ${\text{IP}}_{v}$ = 8.252 eV for the vertical potential, compared with a slightly lower value of ${\text{IP}}_{a}$ = 8.105 eV for the adiabatic potential. The IP values show that the energy required to extract an electron from the HOMO level is high, giving the styrene molecule good stability and reactivity. Concerning EA, it is used to estimate the energy barrier to be crossed for the injection of charge carriers into an organic material [88,89]. For adiabatic and vertical EA, we obtained values of ${EA}_{a}$ = −0.532 eV and ${EA}_{v}$ = −0.727 eV, respectively, the larger value being that of the adiabatic model.

**Figure 2.**
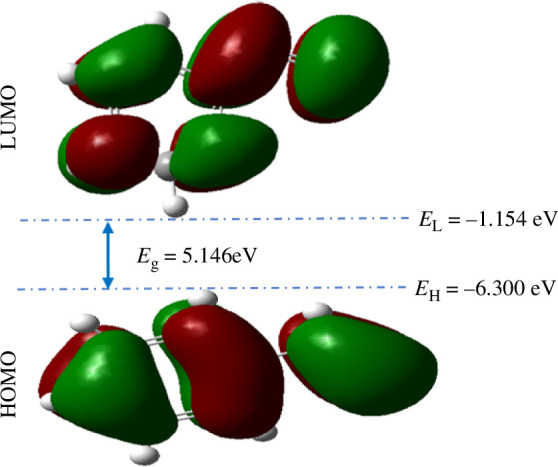
Frontiers molecular orbitals of styrene monomer, obtained using B3LYP.

The charge density distribution of the boundary molecular orbitals in the studied system is shown in figure 2. Regarding the HOMO and LUMO, they consist of negatively charged areas (indicated by the red colour) and positively charged zones (indicated by the green colour). The LUMO exhibits more positively and negatively charged regions than the HOMO. Both the HOMO and LUMO are delocalized throughout the styrene carbon skeleton. The styrene LUMO has many areas of potential interaction with other compounds, both on the phenyl group and on the ethylenic bond, as does the HOMO. The HOMO is located on orbital 28, while the LUMO is located on orbital 29. The energy barrier between these two orbitals is 5.146 eV and represents the band gap. The electronic transition between these two frontier orbitals is considered to be the most achievable and lowest-energy conversion that can enable a charge carrier to move from the HOMO to the LUMO of styrene [33,34] and it corresponds in this case on a π→π* transition. Overall, the styrene monomer has a total of 192 molecular orbitals, of which only 28 are occupied, leaving 164 free. This abundance of molecular orbitals opens up numerous possibilities for electronic transitions. The LUMO and HOMO energies are, respectively, *E*_LUMO_ = −1.154 eV and *E*_HOMO_ = −6.300 eV. Bearing in mind that the HOMO value generally required to be a charge transport polymer (CTP) [90] is between −5.5 and −6.0 eV, we found that the HOMO of styrene is −0.3 eV more than the maximum value required to be a CTP; a difference of 5% from the value that highlights the intrinsic suitability of styrene monomer for charge transport. Based on its HOMO value, functionalization or doping, to name but a few, could easily make styrene a good charge carrier. Relative to other materials available in the literature and recognized to date as potentially very suitable for the design and manufacture of OSC devices, the HOMO in these compounds is on average between −5.0 and −5.65 eV [11,81,84]. The maximum values are approximately 1.3 eV higher than those of styrene, which is an insulator. However, the styrene LUMO is on average 2 eV larger than that of good materials for hole and electron transport. To create effective charge carriers from styrene, it would be necessary to lower this LUMO. Overall, based on its HOMO and LUMO, the styrene monomer is a favourable material for charge transport.

### 3.5. Chemical descriptors of the reactivity

Chemical descriptors of reactivity allow us to discuss the reactivity and stability of the chemical behaviour of a molecule. These investigations can be achieved by the determination of global descriptor parameters such as the chemical potential (*μ*_CP_), chemical hardness (*η*), chemical softness (*S*), electrophilicity index (*ω*), nucleophilicity index (*υ*) and maximum charge transfer (Δ*N*_max_). These parameters for the styrene monomer are summarized in table 6.

**Table 6.** Global reactivity descriptors of styrene monomer in vertical and adiabatic model, obtained using B3LYP.

parameters/methods	vertical	adiabatic
*μ*_CP_ (eV)	−3.763	−3.787
*η* (eV)	4.490	4.319
*S* (eV)^−1^	0.223	0.232
*ω* (eV)	1.577	1.660
*υ* (eV)^−1^	0.634	0.602
∆*N*_max_	0.838	0.877

*μ*_CP_ represents the energy required for an electron to escape from the stable configuration of a molecule [4]. It can be interpreted as the ease with which electrons can abandon one stable molecular system for another [91]. We found that for styrene monomer, *μ*_CP_ = −3.763 eV for vertical evaluation and −3.787 eV for adiabatic assessment. These values are intermediate between those of an insulator and a wide-bandgap semiconductor [2,25]. Based on the chemical potential, the reactivity of styrene monomer is greater than that of MMA monomer, for which *μ*_CP_ = −4.414 eV. Indeed, an increase in the value of *μ*_CP_ leads to an increase in reactivity and a decrease in stability.

Concerning η , we obtained 4.490 and 4.319 eV for the vertical and adiabatic chemical hardness of styrene monomer, respectively. Consequently, despite its insulating nature, styrene monomer may yield electrons to the surrounding medium more readily than MMA monomer [4]. This suggests that styrene functionalization may be more favourable to intramolecular charge transfer, thereby enhancing or preserving the initial semiconducting character of the functionalized chromophore than MMA. Regarding ω , we obtained values of 1.577 and 1.660 eV for the vertical and adiabatic evaluation, respectively. These values show that the investigated system has a good capacity to accept electronic charges from others and remains stable. Styrene’s ability to emit and accept charges leads us to conclude that this molecule is suitable for intramolecular charge transfer processes. Indeed, the more semiconducting an organic material is, the higher the value of its ω . Similarly, low values of the ω index reflect insulating behaviour [2,4,25]. Finally, as regards to ${\Delta N}_{max}$ , we obtained 0.838 and 0.877 eV for vertical and adiabatic assessment, respectively. Compared with the value of 0.825 eV obtained in MMA, this confirms the better charge transport properties in styrene than in MMA.

### 3.6. Linear optical properties

Some selected linear optical parameters of styrene monomer such as *μ*, $\bar{\alpha }$ , Δ*α*, χ_e,_ MR and χ^(1)^) in static mode were evaluated and collected in table 7.

**Table 7.** Dipole moment (*μ*), average polarizability ( $\bar{\alpha }$ ), anisotropy of polarizability (Δ*α*), molar refractivity (MR), first-order susceptibility tensor (*χ* ^(1)^) and average electric susceptibility (*χ*_e_) in static mode of styrene monomer.

parameters/method	B3LYP	ωB97XD
*μ* (D)	0.192	0.149
$\bar{\alpha }$ (×10^−24^ esu)	12.958	12.638
Δ*α* (×10^−24^ esu)	12.504	11.702
MR (esu mol^−1^)	32.691	31.885
χ^(1)^_xx_	3.155	3.211
χ^(1)^_xy_	0.122	0.134
χ^(1)^_yy_	2.153	2.269
χ^(1)^_xz_	0.000	0.009
χ^(1)^_yz_	0.000	0.027
χ^(1)^_zz_	0.876	0.948
χ_e_	2.062	2.143

With regards to μ , a high value is very often synonymous with good optical properties, while a low value often indicates poor optical response [4]. Furthermore, a compound with zero dipole moment (polar molecule) cannot exhibit total static hyperpolarizability, internal electric field or electric polarization [25]. We obtained a value of 0.149 *D* for styrene monomer, a small value which proves that the behaviour of styrene monomer is close to that of polar compounds, but that it can exhibit NLO properties. Comparing with MMA monomer, with values of 1.843 and 1.698 *D* for *cis*- and *trans*-MMA, respectively, we can conclude that the dipole moment of styrene is 13 and 11 times lower than that of *cis*-MMA and *trans*-MMA, respectively.

As far as polarizability is concerned, it provides information on the distribution of electrons in the molecule, and plays an important role in determining the structure and orientation of a material [92]. Materials with low $\bar{\alpha }$ values are weakly polar. We recorded $\bar{\alpha }=12.958\times 10^{-24}esu$ in the styrene monomer, a low value which indicates that styrene is a molecule that deforms very little under the action of an external electric field. Compared with MMA monomer with $\bar{\alpha }=9.007\times 10^{-24}$ and 8.991×10^−24^ esu for *cis*- and *trans*-MMA [4], respectively, we find that styrene monomer offers greater polarizability than MMA. However, owing to their low $\bar{\alpha }$ values, both styrene and MMA can be used to improve materials such as optical fibres [93].

Regarding **χ^(1)^** tensor, we found that styrene monomer exhibits preferential directions of electron displacement. These are the (xx) and (yy) directions along which the material exhibits maximum susceptibility namely $\chi_{xx}^{\left(1\right)}=3.155$ and $\chi_{yy}^{\left(1\right)}=2.153$ . By contrast, the quasi-plane structure of styrene results in a zero response in the (xz) and (yz) directions and a weak response in the (zz) direction with $\chi_{zz}^{\left(1\right)}=0.876$ . Styrene monomer has an average susceptibility *χ*_e_ = 2.062, a value one unit lower than that of some organic broadband semiconductors, which have a first-order average susceptibility of between three and four [2]. This macroscopic response ability of styrene confirms the predictions of a good candidate material for the functionalization of chromophores for applications in nonlinear optics. Finally, directional analysis of linear optical behaviour in terms of polarizability and susceptibility shows that styrene monomer is anisotropic. We obtained a value Δα = 12.504 × 10^−24^ esu, a value at least two times higher than that of MMA monomer [4]. Finally, styrene monomer exhibits a molar refractivity of MR = 32.691 esu mol^−1^.

### 3.7. Nonlinear optical properties

#### 3.7.1. Nonlinear optical properties in static mode

Some NLO properties of our styrene monomer were evaluated in static mode using ωB97XD functional. Thus, the values of β_T_, $\bar{\gamma }$ , $\chi_{T}^{\left(2\right)}$ and $\chi_{T}^{\left(3\right)}$ are presented in table 8.

**Table 8.** First total hyperpolarizability (*β*_T_), averaged second hyperpolarizability ( $\bar{\gamma }$ ), second-order total susceptibility ( $\chi_{T}^{\left(2\right)}$ ) and third-order total susceptibility ( $\chi_{T}^{\left(3\right)}$ ) in static mode of styrene monomer.

parameters/method	ωB97XD
*β*_T_ (×10^−30^ esu)	0.587
$\bar{\gamma }$ (×10^−36^ esu)	4.980
$\chi_{T}^{\left(2\right)}$ (pm V^−1^)	1.658
$\chi_{T}^{\left(3\right)}$ (×10^−22^ m^2^ V^−2^)	1.075

With regards to first-order hyperpolarizability in the static regime, we obtained a value of *β*_T_ = 0.587 × 10^−30^ esu, a value that highlights the absence of centrosymmetry in styrene and the existence of a first-order microscopic response sufficient for the existence of NLO behaviour in styrene monomer. Comparing this value with that of MMA where *β*_T_ = 0.153 × 10^−30^ esu, we see that styrene monomer is 3.83 times better than MMA. It could therefore be more suitable than MMA for the functional design of new NLO materials. With respect to the second-order hyperpolarizability, we obtained a value of $\bar{\gamma }=4.980\times 10^{-36}\;esu$ , an insufficient value to consider styrene as a good material for NLO in the static regime. However, it is 1.66 times higher than that of the monomer MMA where $\bar{\gamma }=3.000\times 10^{-36}\;esu$ .

Concerning the optical susceptibility, we obtained second- and third-order values in the static regime $\chi_{T}^{\left(2\right)}=1.658\;\;pmV^{-1}$ and $\chi_{T}^{\left(3\right)}=1.075\times 10^{-22}\;m^{2}V^{-2}$ , respectively. We note that at third order, styrene and MMA monomers offer almost the same behaviour, while at second order, the value for styrene is three times greater than that for MMA ( $\chi_{T}^{\left(2\right)}$ = 0.503 pmV^−1^).

#### 3.7.2. Pockel’s electro-optic and direct current Kerr effects

The interaction between an organic material and a high-intensity laser source gives rise to several nonlinear optical effects that depend on the frequency and phase used by the laser. These include second harmonics generation (SHG) [24] and multiphoton absorption [94]. However, we can also distinguish two important effects which occur in the dynamic regime and which allow us to characterize the response of the material to a specific frequency [24,91,94]. These are the Pockel’s electro-optic effect( EOPE), which occurs for frequency (−*ω*;*ω*,0) and is related to β , and the direct current Kerr (DC-KERR) effect, which occurs for frequency (−*ω*;*ω*,0,0) and is related to γ . The microscopic and macroscopic Pockel and KERR responses of styrene monomer were assessed and summarized in table 9.

**Table 9.** EOPE first total hyperpolarizability ( $\beta_{EOPE}\left(-\omega ;\omega ,0\right)$ ), DC-KERR averaged second hyperpolarizability ( ${\overset{-}{\gamma }}_{DC-KERR}\;\left(-\omega ;\omega ,0,0\right)$ ), EOPE second-order total susceptibility ( $\chi_{T}^{EOPE}\left(\omega \right)$ ) and DC-KERR third-order total susceptibility ( $\chi_{T}^{DC-KERR}\left(\omega \right)$ ) of styrene monomer using ωB97XD functional, obtained at wavelength λ = 1064 nm.

parameters/molecules	styrene	MMA [4]
$\beta_{EOPE}\left(-\omega ;\omega ,0\right)$ (×10^−30^ esu)	0.699	0.153
${\overset{-}{\gamma }}_{DC-KERR}$ (-ω;ω,0,0) (×10^−36^ esu)	5.509	3.000
$\chi_{T}^{EOPE}\left(\omega \right)$ (pm V^-1^)	1.974	0.510
$\chi_{T}^{DC-KERR}\left(\omega \right)$ (×10^−22^ m^2^ V^−2^)	2.609	0.856

The EOPE can be understood as the ability of a dielectric material to modify its polarization owing to a change in birefringence when a strong electric field is applied to it. A material with a high response can then find many applications in light modulation and switching devices, owing to its ability to modify the propagation of light within it, in the presence of an electric field. We determined the EOPE effect by evaluating $\beta_{EOPE}\left(-\omega ;\omega ,0\right)$ and $\chi_{T}^{EOPE}\left(\omega \right)$ at the usual wavelength of 1064 nm. From the values in table 9, we obtain $\beta_{EOPE}\left(-\omega ;\omega ,0\right)=0.699\;\times 10^{-30}\;esu$ and $\chi_{T}^{EOPE}\left(\omega \right)=1.974\;pmV^{-1}$ , values which highlight the existence of the EOPE in styrene monomer. Compared with MMA, styrene monomer is therefore better suited to the functionalization of materials suitable for the electro-optical Pockel effect.

The DC-KERR effect, meanwhile, is related to γ and can be considered as the material’s ability to change its refractive index as a function of incident light intensity. A high value of ${\overset{-}{\gamma }}_{DC-KERR}$ allows us to postulate potential applications of the material in the manufacture of high-speed optical modulators, while a high value $\chi_{T}^{DC-KERR}\left(\omega \right)$ allows us to postulate potential applications in the generation of optical harmonics. For styrene monomer, we obtained ${\overset{-}{\gamma }}_{DC-KERR}\left(-\omega ;\omega ,0,0\right)=5.509\times 10^{-36}\;esu$ and $\chi_{T}^{DC-KERR}\left(\omega \right)=2.609\times 10^{-22}\;m^{2}V^{-2}$ . Styrene monomer is therefore suitable for modulating its refractive index as a function of the intensity of the electrical excitation signal. However, its optical rigidity induces weak NLO responses, owing to its low intramolecular charge transfer.

#### 3.7.3. Second harmonics generation and third harmonics generation

To assess the ability of styrene to generate specific frequency mixing processes such as SHG and third harmonics generation (THG), the first-order (*β*(−2 *ω*;*ω*,*ω*)) and second-order (γ(−2 *ω*;*ω*,*ω*,0)) as well as the resulting optical susceptibilities $\chi_{T}^{SHG}\left(-2\omega ;\omega ,\omega \right)$ and $\chi_{T}^{THG}\left(-2\omega ;\omega ,\omega \right)$ at the wavelength of 1064 nm were determined and reported in table 10.

**Table 10.** Some selected components of the frequency-dependent first-order hyperpolarizability $\beta \left(-2\omega ,\omega ,\omega \right)$ (in 10^−30^ esu) and second-order hyperpolarizability $\gamma \left(-2\omega ,\omega ,\omega ,0\right)$ (in 10^−36^ su), second- and third-order total optical susceptibilities $\chi_{T}^{SHG}\left(-2\omega ;\omega ,\omega \right)$ and $\chi_{T}^{THG}\left(-2\omega ;\omega ,\omega \right)$ at λ = 1064 nm.

parameters/method	ωB97XD
*β*_*xxx*_	0.009
*β*_yyy_	−0.121
*β*_zzz_	−0.838
*β*_x_	0.020
*β*_y_	−0.116
*β*_z_	−0.838
$\beta_{T}\left(-2\omega ;\omega ,\omega \right)$	0.847
*γ*_xxxx_	0.161
*γ*_yyyy_	3.382
*γ*_zzzz_	26.323
*γ*_xxyy_	0.299
*γ*_yyzz_	0.505
*γ*_xxzz_	0.581
$\bar{\gamma }\left(-2\omega ;\omega ,\omega ,0\right)$	6.527
$\chi_{T}^{SHG}\left(-2\omega ;\omega ,\omega \right)$ (pm V^−1^)	2.392
$\chi_{T}^{THG}\left(-2\omega ;\omega ,\omega \right)$ (×10^−22^ m^2^ V^−2^)	1.410

Analysis of the *β*(−2 *ω*;*ω*,*ω*) and γ(−2 *ω*;*ω*,*ω*,0) tensors reveals strong anisotropy in the NLO response of styrene monomer. In the case of β , intramolecular charge transfer predominates in the (zzz) direction, in contrast to linear optics, where the response is governed by the (xx) direction. The presence of an external electric field effectively modifies the polarization and topology of the electron pattern. Similarly, analysis of γ reveals a second-order nonlinear response strongly dominated by the *γ*_zzzz_ component with a value of 26.323×10^−36^ esu. For styrene monomer, we obtained $\beta_{T}\left(-2\omega ;\omega ,\omega \right)=0.847\times 10^{-30}\;esu$ and $\bar{\gamma }\left(-2\omega ;\omega ,\omega ,0\right)=6.527\times 10^{-36}\;esu$ . Comparing these values with experimental values for urea $\beta_{T}=2.3\times 10^{-30}$ and $\bar{\gamma }=48\times 10^{-36}esu$ [95], which is the reference molecule for NLO properties, we found that urea has a total first hyperpolarizability 2.71 times higher than styrene monomer. Similarly, styrene monomer has an average second hyperpolarizability at least 7.35 times lower than urea.

Consequently, the studied monomer is not a suitable candidate for devices requiring good NLO properties. On the other hand, compared with the NLO behaviour of MMA, styrene exhibits a β 3.38 times greater than that of MMA, and a γ 1.86 times greater than that of MMA. As a result, styrene monomer is a much better material for harmonic generation than MMA monomer, and could be a better material than MMA for the design of new materials for NLO.

For second- and third-order susceptibilities, we obtained $\chi_{T}^{SHG}\left(-2\omega ;\omega ,\omega \right)=2.392\;\;pmV^{-1}$ and $\chi_{T}^{THG}\left(-2\omega ;\omega ,\omega \right)=1.410\times 10^{-22}\;m^{2}V^{-2}$ . Our value of $\chi_{T}^{SHG}$ is 2.39 times greater than that of quartz ( $\chi_{T}^{SHG}=1pmV^{-1}$ ) [96], which is a reference material for SHG. It can, therefore, be considered a material suitable for SHG. On the other hand, the $\chi_{T}^{THG}=2\times 10^{-22}\;m^{2}V^{-2}$ value of silica [97,98], which is a reference material for THG, is about 1.42 times higher than that of styrene monomer. Styrene monomer is therefore not sufficiently suitable for THG. However, the $\chi_{T}^{THG}$ value of styrene is higher than that of MMA.

#### 3.7.4. Hyper-Rayleigh scattering

Hyper-Rayleigh scattering (HRS) is one of the experimental techniques used to measure the intensity of the incoherently scattered frequency-doubled light generated after the interaction of a laser beam with a chromophore in an isotropic solution [99]. As optical communication networks evolve, the research and development of new compounds with NLO performance is of significant importance for improving this field [100,101]. Prediction of $\beta_{HRS}$ is essential for identifying, developing and improving raw materials suitable for use in optical communications [99], as well as for frequency doubling, ultrafast lasers and fast electro-optical modulation [102]. The value of HRS can be evaluated theoretically using the following equation [102]:


(3.5) $$\beta_{HRS}\left(-2\omega ;\omega ,\omega \right)={\left(\beta_{zzz}^{2}\left(-2\omega ;\omega ,\omega \right)+\beta_{xzz}^{2}\left(-2\omega ;\omega ,\omega \right)\right)}^{1/2},$$

where $\beta_{zzz}^{2}\left(-2\omega ;\omega ,\omega \right)$ and $\beta_{xzz}^{2}\left(-2\omega ;\omega ,\omega \right)$ stand to the orientationally averaged tensor components. On the other hand, the depolarization ratio (DR), which is useful for measuring the dipolar to octupolar contribution of the studied compound, was evaluated as follows:


(3.6) $$DR=\beta_{zzz}^{2}/\beta_{xzz}^{2}.$$

In addition, another important NLO parameter is the degenerate four-wave mixing response ( $\gamma^{DFWM}$ ) and the nonlinear quadratic refractive index (*n*_2_), which allows us to discuss potential optoelectronic applications of the material as a wavelength converter or optical pulse modulator when the material exhibits large index values [103]. $\gamma^{DFWM}$ was assessed using the formula:


(3.7) $$\gamma^{DFWM}\left(\omega \right)=\gamma^{DFWM}\left(-\omega ;\omega ,-\omega ,\omega \right)\approx \frac{1}{3}\gamma \left(-2\omega ;\omega ,\omega ,0\right)+\gamma \left(-\omega ;\omega ,0,0\right)-\frac{1}{3}\bar{\gamma \;}\;$$

and *n*_2_ using the following equation:


(3.8) $$n_{2}\left(\omega \right)=\frac{3}{4\varepsilon_{0}cn_{0}^{2}}\chi^{DFWM}\left(\omega \right)\;with\;n_{0}^{2}=1+\chi_{tot}^{\left(1\right)},$$

where *n*_0_ is the linear refractive index and *c* the speed of light in vacuum. The values of these parameters are listed in table 11.

**Table 11.** Frequency-dependent NLO response of styrene monomer, obtained at *λ* = 1064 nm: HRS response $\beta_{HRS}\left(-2\omega ;\omega ,\omega \right)$ , $DR\left(-2\omega ;\omega ,\omega \right)$ , degenerate four-wave mixing of the third-order response $\gamma^{DFWM}$ and nonlinear refractive index $n_{2}$ .

parameters/method	ωB97XD
$\beta_{HRS}\left(-2\omega ;\omega ,\omega \right)$ (×10^−30^ esu)	0.357
$DR\left(-2\omega ;\omega ,\omega \right)$	4.556
$\gamma^{DFWM}\left(\omega \right)$ (×10^−36^ esu)	6.024
$\chi^{DFWM}\left(\omega \right)$ (×10^−22^ m^2^ V^−2^)	1.891
$n_{2}\left(\omega \right)$ (×10^-20^ m^2^W^−1^)	1.748

The values in table 11 show that $\beta_{HRS}=0.357\times 10^{-30}esu$ , a non-zero value that confirms the existence of first-order NLO activity in styrene. This activity is half that of *trans*-MMA and slightly lower than that of *cis*-MMA [4]. The DR obtained is 4.556, a value higher than 4.5, giving styrene a dipolar contribution.

With regards to the DFWM response, we obtained $\gamma^{DFWM}=6.024\times 10^{-36}esu$ , a value twice that of MMA. Styrene monomer has a greater capacity for second-order NLO response than first-order NLO response. Indeed, as a third-order susceptibility derived from $\gamma^{DFWM}$ , we obtained a value $\chi^{DFWM}\left(\omega \right)=1.891\times 10^{-22}m^{2}V^{-2}$ , smaller than that of silica [97,98], but still higher than that of MMA.

Finally, we obtained $n_{2}\left(\omega \right)=1.748\times 10^{-20}\;m^{2}W^{-2}$ , a value 36% lower than that obtained in fused silica $n_{2}=\left(2.74\pm 0.17\right)\times 10^{-20}\;m^{2}/W$ [104], and 32% lower than that obtained experimentally in the CS_2_ liquid $n_{2}=\left(2.6\pm 0.6\right)\times 10^{-18}\;m^{2}/W$ [105], commonly used as reference material for DFWM . The $n_{2}$ value of styrene monomer is at least 40% higher than that of the two MMA isomers. Styrene should therefore be more suitable for the design of new degenerate four-wave mixing materials than MMA.

### 3.8. Thermodynamic properties

The thermodynamic stability of an organic system is a key criterion that it must meet to find applications in industry or to react with other compounds. We have evaluated some key parameters of the thermodynamic activity of styrene monomer and the results are given in table 12.

**Table 12.** Thermodynamic properties of styrene monomer.

parameters/methods	B3LYP	ωB97XD
EE (×10^3^ kcal mol^−1^)	−194.357	−194.282
ZPVE (kcal mol^−1^)	83.326	84.167
H (×10^3^ kcal mol^−1^)	−194.269	−194.193
G (×10^3^ kcal mol^−1^)	−194.293	−194.218
*E*_Th_ (kcal mol^−1^)	87.596	88.378
Cv (cal mol^−1 ^K)	26.134	25.859
*S* (cal mol^−1 ^K)	82.898	81.721

The Gibbs free energy (*G*) is a fundamental criterion for the thermodynamic stability of a system. The lower the value for of compound, the more stable it is [2]. Another important aspect of *G*, is its ability to predict whether or not a system can react with another system. We obtained a value of −194.293×10^3^ and −194.218×10^3^ kcal mol^−1^ using B3LYP and ωB97XD, respectively. These negative values give styrene thermodynamic stability and the ability to react with other compounds. It can also be noted that styrene is less stable and slightly more reactive than MMA [4]. With regards to zero-point vibrational energy (ZPVE), we obtained a value of 83.326 kcal mol^−1^ through B3LYP and 84.167 kcal mol^−1^ through ωB97XD, values 8% higher than those for MMA. As far as thermal energy (*E*_Th_) is concerned, this is the kinetic energy of the microscopic agitation of a system, owing to the disordered agitation of its atoms and molecules [2]. For styrene monomer, we obtained *E*_Th_ = 87.596 and 88.378 kcal mol^−1^ using B3LYP and ωB97XD, respectively, relatively low values owing to the small size of the styrene molecule, which favours its stability. Nevertheless, these values are higher than those for MMA by approximately 7%. For heat capacity, we obtained *C*_v_ = 26.134 kcal mol^−1^ using B3LYP and C_v_ = 25.859 kcal mol^−1^ using ωB97XD, values 5% lower than those for MMA. These values show that styrene’s ability to withstand any increase in temperature is low. However, the values are sufficient to guarantee good thermal resistance. Regarding entropy (*S*), we obtained a value of 82.898 cal mol^−1 ^K based on B3LYP and 81.721 cal mol^−1 ^K based on ωB97XD, i.e. 5% more intramolecular disorder than in MMA. Finally, for enthalpy (*H*), another key parameter of thermodynamic stability, we obtained negative values *H* = −194.269 × 10^3^ kcal mol^−1 ^and *H* = −194.193 × 10^3^ kcal mol^−1^ by B3LYP and ωB97XD, respectively, values which again suggest the thermodynamic stability of styrene.

Finally, the thermodynamic parameters of styrene show that it is stable and able to react with other compounds to form new ones. It can, therefore, be used to functionalize chromophores and other materials. In addition, it was found to offer virtually the same thermodynamic performance as MMA.

### 3.9. Ultraviolet-visible spectroscopy analysis

#### 3.9.1. Ultraviolet-visible spectroscopy absorption spectra analysis

**Figure 3.**
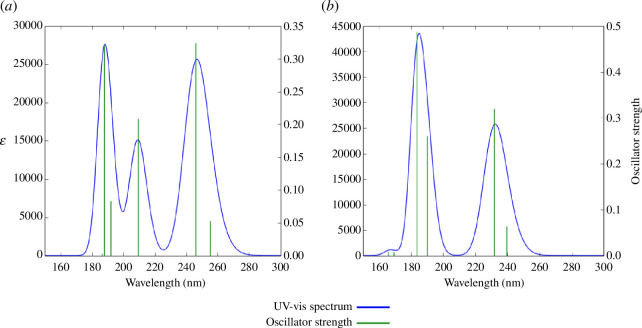
TD-DFT absorption spectra of styrene monomer. (*a*) B3LYP and (*b*) 𝜔B97XD.

In this section, we evaluate the behaviour of styrene monomer in excited states after photon absorption, by determining the first allowed singlet-singlet excitation energies. The TD-DFT/B3LYP and TD-ωB97XD methods were used for this purpose. The absorption spectra of styrene are shown in figure 3, while the maximum absorption wavelength (λ), the first six electronic excitation energies (*E*), the oscillation strength (*f*) and their main electronic transitions are presented in table 13.

**Table 13.** Singlet–singlet permitted excitation energies (*E*), maximum absorption wavelength (*λ*), oscillator strength (*f*), efficacity of absorption (*η*) and major electronic contribution to transitions of absorption spectra of styrene monomer obtained using TD-B3LYP and TD-ωB97XD.

excited state/parameters	*E* (eV)	*λ*(nm)	*f*	*η*	major contribution
TD-B3LYP					
1	4.860	255.10	0.053	0.115	H−1 → L (42%), H → L (16%), H → L+1 (41%)
2	5.039	246.04	0.324	0.526	H−1 → L (15%), H → L (80%)
3	5.927	209.19	0.209	0.381	H−1 → L (39%), H → L+1 (51%)
4	6.460	191.92	0.083	0.174	H−2 → L (65%), H−1 → L+1 (17%), H → L+3 (16%)
5	6.608	187.62	0.321	0.523	H−1 → L+1(63%), H → L+3 (30%)
6	6.650	186.43	0.003	0.006	H → L+2 (98%)
TD-ωB97XD					
1	5.174	239.62	0.063	0.136	H−1 → L (37%), H → L (19%), H → L+1 (39%)
2	5.353	231.62	0.320	0.521	H−1 → L (14%), H → L (75%)
3	6.523	190.08	0.261	0.452	H−1 → L (43%), H → L+1 (51%)
4	6.752	183.61	0.488	0.675	H−1 → L+1 (78%)
5	7.327	169.21	0.008	0.017	H−3 → L (61%), H−2 → L (24%)
6	7.474	165.89	0.009	0.021	H−4 → L (16%), H −3→ L(26%), H−2 → L (36%), H → L+3 (13%)

Figure 3 and table 13 reveal some differences depending on the functional used. Using the ωB97XD functional, two maximum absorption peaks were clearly identified. The low-intensity peak is located at wavelength *λ* = 231.62 nm with an oscillator strength of 0.320 and an absorption efficiency of 0.521. This transition corresponds to an electron jump from HOMO−1 to LUMO with 14% contribution and from HOMO to LUMO with 75% contribution.

The maximum absorption peak is located at wavelength λ = 183.61 nm with maximum oscillator strength and absorption efficiency of 0.488 and 0.675, respectively. This peak is attributed to an electron jump from HOMO−1 to LUMO+1 with 78% contribution. As far as the B3LYP functional is concerned, we have an average absorption band at λ = 209.19 nm and two intense absorption bands at λ = 246.04 and λ = 187.62 nm. Finally, whatever the functional used, like MMA [4], styrene absorbs in the ultraviolet. Both compounds thus appear as potential candidates for the manufacture of ultraviolet (UV) sensors.

#### 3.9.2. Ultraviolet-visible spectroscopy emission spectra analysis

**Figure 4.**
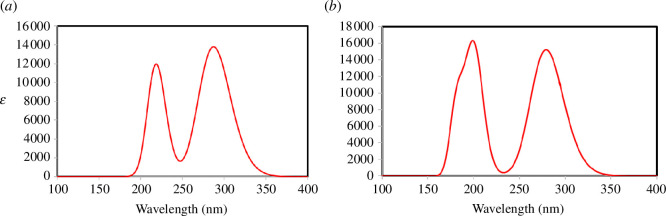
Theoretical (*a*) TD-DFT/B3LYP and (*b*) TD-DFT/ωB3LYP emission spectra of styrene monomer.

Emission properties are important parameters for suggesting applications of organic materials in display devices, as well as in light sources and in the manufacture of OLEDs. In this section, the emission spectrum of styrene and some important properties such as Stokes shift and radiative lifetime (*τ*) have been determined. The emission spectrum is shown in figure 4, while the emission properties are reported in table 14.

**Table 14.** Fluorescent emission wavelength (λ), fluorescence energy (*E*_Flu_), oscillator strength (*f*), Stokes shift (Δλ) and radiative lifetime ( τ ) of styrene monomer.

parameters	TD-B3LYP	TD-ωB97XD
*λ* (nm)	287.99	200.90
*E*_Flu_(eV)	4.315	6.201
*f*	0.330	0.371
Δ*λ* (nm)	41.95	17.29
τ (ns)	3.863	1.662

The Stokes shift ( Δλ is the difference between a molecule’s maximum emission wavelength and its maximum absorption wavelength [106]. It indicates the relationship between the structure and properties of fluorescent molecules between their ground and excited states [107]. We have calculated the Stokes shift using the following equation [108]:


(3.9) $$\Delta \lambda =\lambda^{em}-\lambda^{abs}.$$

Δλ of styrene monomer given by the TD-ωB97XD method is 17.29 nm, a value seven times higher than that of the *cis* form of MMA, a result which allows us to conclude that styrene monomer is more chemically stable than *cis*-MMA. On the other hand, calculation by the TD-B3LYP method led to Δ*λ* = 41.95 nm, a larger value than that calculated by TD-ωB97XD. Comparing this value obtained by the TD-B3LYP method with that of *cis*-MMA, it appears that the stability of styrene is three times greater than that of MMA, and that the value of the (Δ*λ*) depends on the functional used. The low Stokes shift values of styrene monomer limit its use as a potential material for the manufacture of solar cell devices [93].

The emission spectra in figure 4 show two prominent emission bands, regardless of the method used. Using TD-ωB97XD, the fluorescent emission wavelength is 200.90 nm and is attributed to the HOMO−1 → LUMO transition with 44% contribution and to the HOMO → LUMO+1 transition with 50% contribution. In contrast, using TD-B3LYP, the fluorescent spectrum of styrene monomer shows a maximum emission wavelength of 287.99 nm, a difference of 87.09 nm compared with TD-ωB97XD. The transition involved in the maximum emission is essentially a HOMO → LUMO transition with 94% contribution and an oscillator strength of 0.330. According to the present results, styrene monomer emits only in the ultraviolet.

The time a fluorescent molecule remains in an excited state in the absence of a non-radiative transition is called its radiative lifetime [58]. It is defined (in arbitrary units) as follows [58,89,107]:


(3.10) $$\tau =\frac{c^{3}}{2(E_{flu}{)}^{2}f},$$

where *E*_flu_ is the fluorescence energy, *f* the oscillator strength and *c* the speed of light. We obtained for styrene monomer *τ* = 3.863 ns using TD-B3LYP and *τ* = 1.662 ns using TD-ωB97XD.

### 3.10. Natural bond orbital analysis

#### 3.10.1. Natural bond charge analysis

**Figure 5.**
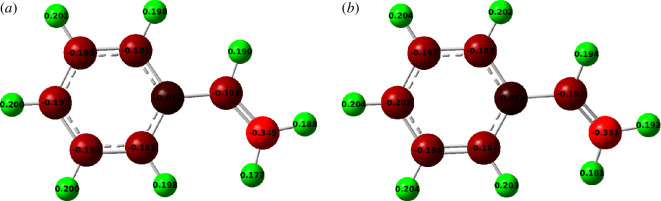
NBO charges of styrene monomer (*a*) B3LYP and (*b*) ωB97XD.

According to several authors [17,91,94,109], NBO analysis enables us to understand the direction of charge transfer taking place in the molecule, and to identify the atoms or groups of atoms that play the roles of donor and acceptor. NBO calculations were conducted using the two B3LYP and ω B97XD functionals, the results are depicted in figure 5.

Using B3LYP, the NBO charges on the styrene monomer range from −0.345 e to +0.201 e. The maximum positive charge of +0.201 e is obtained on the H7-labelled hydrogen atom of the phenyl ring involved in the C1–H7 bond.

We found that all hydrogen atoms have an average positive charge of +0.194 e. It follows that the charges leave all the hydrogen atoms towards the carbon atoms, which are then all negatively charged. The system’s highest negative charge in absolute value −0.345 e is obtained on the C14-labelled carbon in the ethylene group [110]. The system’s highest negative charges are located on the carbon of the phenyl group. Thus, the probable direction of charge displacement is from the phenyl group, as charge donor, to ethylene, as receptor. Finally, all atoms are involved in intramolecular charge transfer, which helps us to understand the origin of the optical and transport properties observed in styrene monomer. The results obtained using ωB97XD are quite similar to those of B3LYP, even if slightly higher, and the overall behaviour observed on intramolecular charge transfer is the same.

#### 3.10.2. Second order Fock matrix analysis

In NBO analysis, the second-order Fock matrix is a means of investigating the donor-acceptor interaction. The higher the degree of conjugation, the more stability of the molecule will be ensured [111]. The stabilization energy is a key parameter for this analysis. A higher value of *E*(2) indicates a strong interaction between the donor orbital, and the acceptor orbital, leading to a higher degree of electron delocalization [112]. Given a donor (*i*) and an acceptor (*j*), the diagonal members related to the orbital energies values of acceptor and donor $\varepsilon_{j}$ and $\varepsilon_{i}$ , respectively; the stabilization energy *E*(2) can be obtained through equation (3.11) [112–114]


(3.11) $$E\left(2\right)=\Delta E_{ij}=q_{i}\frac{(F_{i}{,}_{j}{)}^{2}}{\varepsilon_{j}-\varepsilon_{i}},$$

where $q_{i}$ refers to the orbital occupancy and $F_{ij}$ represents the elements of the Fock matrix. The results of styrene monomer bond interaction analysis are reported in table 15.

**Table 15.** NBO second-order perturbation theory analysis of Fock matrix of styrene monomer, obtained using the B3LYP method.

donor NBO (*i*)	type	acceptor NBO (*j*)	type	*E*(2) kcal mol^−1^	*E*(*i*)*−E*(*j*) arbitrary units	*F*(*i*,*j*) arbitrary units
C1–C2	σ(−1)	C1–C6	σ*(1)	3.17	1.28	0.057
C1–C2	σ(−2)	C3–C4	σ*(2)	19.9	0.29	0.068
C1–C2	σ(−2)	C5–C6	σ*(2)	20.07	0.29	0.068
C1–C6	σ(−1)	C1–C2	σ*(1)	3.15	1.28	0.057
C1–C6	σ(−1)	C5–C6	σ*(1)	3.52	1.26	0.06
C1–C6	σ(−1)	C5–C12	σ*(1)	3.32	1.18	0.056
C1–H7	σ(−1)	C2–C3	σ*(1)	3.79	1.09	0.057
C1–H7	σ(−1)	C5–C6	σ*(1)	4.16	1.08	0.06
C2–C3	σ(−1)	C3–C4	σ*(1)	3.12	1.28	0.057
C2–H8	σ(−1)	C1–C6	σ*(1)	3.85	1.1	0.058
C2–H8	σ(−1)	C3–C4	σ*(1)	3.81	1.1	0.058
C3–C4	σ(−1)	C2–C3	σ*(1)	3.11	1.27	0.056
C3–C4	σ(−1)	C4–C5	σ*(1)	3.71	1.27	0.061
C3–C4	σ(−1)	C5–C12	σ*(1)	3.59	1.18	0.058
C3–C4	σ(−2)	C1–C2	σ*(2)	19.98	0.28	0.068
C3–C4	σ(−2)	C5–C6	σ*(2)	19.88	0.29	0.068
C3–H9	σ(−1)	C1–C2	σ*(1)	3.74	1.1	0.057
C3–H9	σ(−1)	C4–C5	σ*(1)	4.25	1.08	0.061
C4–C5	σ(−1)	C3–C4	σ*(1)	3.46	1.27	0.059
C4–C5	σ(−1)	C5–C6	σ*(1)	4.07	1.25	0.064
C4–C5	σ(−1)	C5–C12	σ*(1)	3.17	1.17	0.054
C4–H10	σ(−1)	C2–C3	σ*(1)	3.91	1.09	0.058
C4–H10	σ(−1)	C5–C6	σ*(1)	4.31	1.08	0.061
C5–C6	σ(−1)	C1–C6	σ*(1)	3.27	1.27	0.058
C5–C6	σ(−1)	C4–C5	σ*(1)	4	1.25	0.063
C5–C6	σ(−2)	C1–C2	σ*(2)	20.93	0.28	0.069
C5–C6	σ(−2)	C3–C4	σ*(2)	19.31	0.28	0.067
C5–C6	σ(−2)	C12–C14	σ*(2)	14.8	0.29	0.063
C5–C12	σ(−1)	C4–C5	σ*(1)	3.04	1.22	0.054
C5–C12	σ(−1)	C12–C14	σ*(1)	3.4	1.31	0.06
C6–H11	σ(−1)	C1–C2	σ*(1)	3.79	1.09	0.058
C6–H11	σ(−1)	C4–C5	σ*(1)	4.46	1.08	0.062
C12–H13	σ(−1)	C4–C5	σ*(1)	4.67	1.07	0.063
C12–H13	σ(−1)	C14–H15	σ*(1)	4.84	0.94	0.06
C12–C14	π(−1)	C5–C12	σ*(1)	3.6	1.23	0.06
C12–C14	π(−2)	C5–C6	σ*(2)	11.32	0.31	0.057
C14–C15	σ(−1)	C12–H13	σ*(1)	4.86	0.96	0.061
C14–H16	σ(−1)	C5–C12	σ*(1)	6.88	1.01	0.074
C4	CR(−1)	C5	RY*(2)	2.32	11.57	0.146
C14	CR(−1)	C12	RY*(2)	2.67	11.23	0.155
C1–C2	σ*(−2)	C1	RY*(3)	2.65	0.58	0.086
C1–C2	σ*(−2)	C2	RY*(3)	2.77	0.57	0.087
C5–C6	σ*(−2)	C6	RY*(3)	3.4	0.6	0.094

We restricted ourselves mainly to interactions offering the highest stabilization energies (*E*(2) ≥ 3 kcal mol^−1^), and a few specific bonds with values of *E*(2) ≥ 2 kcal mol^−1^. We note the existence of several types of bonds in styrene including bonds: σ → σ*, π → σ*, π → RY*, CR → RY*, σ → RY*, σ*→ RY* but also σ → π *. The most predominant bond is σ → σ*. The main observation made is that the interactions between the C5−C6 and C1−C2 and also between the C1−C2 and C5−C6 bonds of the phenyl ring, lead to two σ→ σ* transitions, which, respectively display *E*(2) = 20.93 and *E*(2) = 20.07 kcal mol^−1^. These two values are the highest stabilization energy of the molecular system. It can be deduced that these interactions within the phenyl group are those which contribute most significantly to the overall stability and cohesion of the styrene monomer. However, these two bonds alone do not ensure the monomer’s stability and reactivity. In fact, there are several other high stabilization energies close to the above maximum values. These include 19.98, 19.90, 19.88 and 19.31 kcal mol^−1^ resulting, respectively, from interactions between C3−C4 and C1−C2 bonds and vice versa; and from interaction between C3−C4 and C5−C6 bonds and vice versa. All these bonds are of the σ → σ* type. The stability of the styrene monomer is essentially guaranteed by the interactions between the σ → σ* bonds within the phenyl group.

The system displays a number of π → σ* and σ → π* transitions, including those resulting from the interaction between the C12−C14 and C5−C6 bond, with energy *E*(2) = 11.32 kcal mol^−1^. It is the most important of the π → σ* transitions. Similarly, an energy *E*(2) = 14.8 kcal mol^−1^ is derived from the interaction between C5−C6 and C12−C14 for the most important σ → π* type transition. The two bonds, although second-neighbours, are quite strongly conjugated, with significant stabilization energies.

The C5−C6 bond interacts with the Ryberg state of the C6 carbon, giving rise to a stabilization energy of 3.4 kcal mol^−1^ and a σ*→ RY* type transition. A CR → RY* transition results from interaction between the Rydberg states of carbons C14 and C12, and an energy *E*(2) = 2.67 kcal mol^−1^ accompanies the transition.

Overall our intermolecular interactions, our molecular system has several σ → σ* transitions which are more favourable to a low delocalization of electrons that leads to weak NLO properties and an insulator electrical behaviour that has been previously observed.

### 3.11. Molecular electrostatic potential

**Figure 6.**
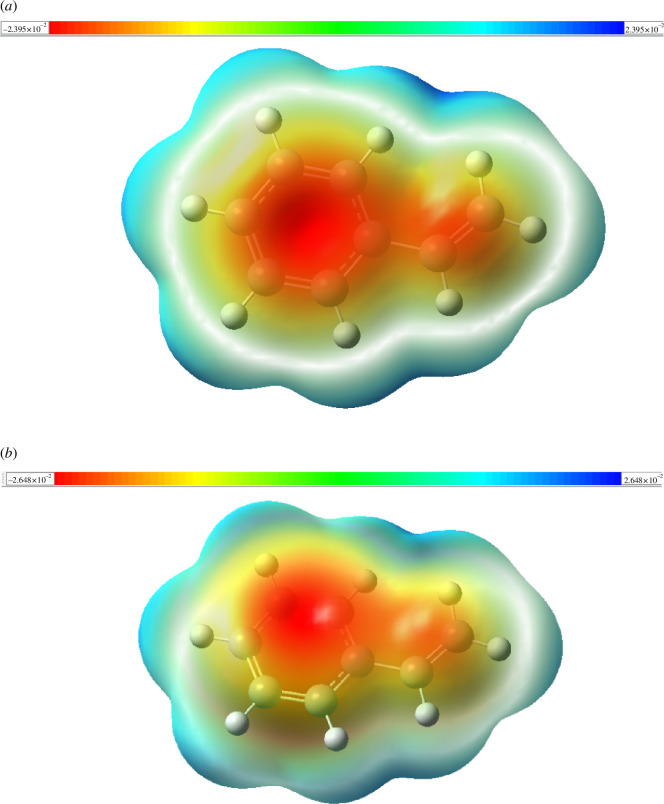
MEP of styrene monomer: (*a*) B3LYP and (*b*) 𝜔𝐵97𝑋D.

The molecular electrostatic potential (MEP) surface is a hypothetical surface that maps over the molecular geometries, allowing us to visualize variably charged regions of a molecule [115]. MEP is also commonly used to study molecule polarity and identify reactive sites [116,117]. In fact, MEP highlights the nucleophilic and electrophilic sites of the molecule [118,119]. In figure 6, we present the MEP of the styrene monomer obtained using B3LYP and ωB97XD. On a MEP surface, the predominant colours are red and blue, indicating electron-withdrawing and electron-donating moieties, respectively. The general order of potential increase is: red < orange < yellow < green < blue [91]. The most electron-rich regions appear in red, while the red, orange and yellow regions correspond to a negative potential [120].

Figure 6 shows that the most electron-rich areas of the styrene monomer are located in the phenyl ring and on the ethylene bond, whatever the functional used. Electrostatic potential values obtained using B3LYP range from −2.395 × 10⁻² to 2.395 × 10⁻² esu, while values obtained with ωB97XD range from −2.648 × 10⁻² to 2.648 × 10⁻² esu. The use of the long-range functional ωB97XD slightly influences the electrostatic potential values of the styrene monomer. Areas where electrons are weakest are shown in blue [120]. These regions have a positive potential and appear as preferred sites for nucleophilic attack [88]. In styrene monomer, we found that electrophilic sites are concentrated on hydrogen atoms, while nucleophilic sites are located along the numerous carbon-carbon bonds present in the monomer.

## 4. Conclusion

In this work, we studied the electronic structures of styrene monomers using DFT and TD-DFT methods. Based on the results of the geometric optimization, we found that the two functionals used, B3LYP and ωB97XD, accurately optimize the geometric structures with bond lengths and angles consistent with the experimental results available in the literature. At the optoelectronic level, styrene exhibits a relative dielectric constant value of 3.062 , making it a good insulator, while we have n=1.750 , which is 13% higher than the experimental index of 1.544 at 25°C. This refractive index value reflects a transparency comparable to that of glass, the reference material. The electronic findings show that the energy gap value is *E*_g_ = 5.146 eV, giving styrene an insulating character. The high IP (8.252 eV) gives styrene monomer good stability and reactivity. Styrene is also thermodynamically stable thanks to its negative *G* value. Based on the findings of UV-visible spectroscopy analysis, styrene absorbs and emits mainly in the UV, at wavelengths of 183.61 and 200.90 nm, respectively. In addition, the Stokes shift of 41.95 nm is low and reduces potential applicability in devices such as solar cells. With regard to linear optical properties, we found that compared with MMA, styrene monomer has a very low μ of 0.192 *D*, but exhibits better linear optical properties such as $\overset{-}{\alpha }$ and Δα . From the transport properties, we obtained electron and hole reorganization energies of 0.393 and 0.295 eV, respectively, meaning that styrene monomer is more favourable to hole transport than electron transport. In terms of nonlinear optics, styrene has a non-zero value of *β*_HRS_, which confirms the existence of first-order NLO activity in styrene, while the DR gives styrene a dipolar contribution. In fact, showing that SHG response of styrene is 2.39 times greater than that of quartz, which is a reference material for SHG. Styrene is also more suitable for the potential generation of THG than MMA. For EOPE, styrene is ideally suited to the functional design of materials suitable for the electro-optical Pockel effect. Indeed, its EOPE susceptibility is almost times higher than that of MMA, making it a better functionalizer than MMA. Finally, compared with MMA, we can theoretically conclude that styrene monomer has good intrinsic potential for the functionalization-based design of new materials for optoelectronics, transparent organic semiconductors, NLO applications and charge transport.

## Data Availability

All input and Gaussian checkpoint file as well as output are available from the Dryad Digital Repository [110].
